# Stimulatory Effect of *Delonix regia* Flower Extract in Protecting *Syzygium cumini* Seedlings from Salinity

**DOI:** 10.3390/plants14060875

**Published:** 2025-03-11

**Authors:** Abdullah H. Alayafi, Abeer A. Dahab, Abdel-Nasser A. El-Sheshtawy, Ashutosh Sharma, Abeer Elhakem, Samah M. Youssef, Rasha S. El-Serafy

**Affiliations:** 1 Department of Agriculture, Faculty of Environmental Sciences, King Abdulaziz University, Jeddah 12619, Saudi Arabia; alayafi06@hotmail.com; 2 Medicinal and Aromatic Plants Research Department, Horticulture Research Institute, Agricultural Research Center, Giza 12619, Egypt; dahab.abeer1612@arc.sci.eg; 3 Environment and Bio-Agriculture Department, Faculty of Agriculture, Al-Azhar University, Cairo 11884, Egypt; 4 Faculty of Agricultural Sciences, DAV University, Jalandhar 144012, Punjab, India; ashutosh10110@davuniversity.org; 5 Department of Biology, College of Sciences and Humanities, Prince Sattam Bin Abdulaziz University, Al-Kharj 11942, Saudi Arabia; a.elhakem@psau.edu.sa; 6 Horticulture Department, Faculty of Agriculture, Fayoum University, Fayoum 63514, Egypt; smy00@fayoum.edu.eg; 7 Horticulture Department, Faculty of Agriculture, Tanta University, Tanta 31527, Egypt

**Keywords:** plant extract, salinity, jamun, oxidative burst, reactive oxygen species, antioxidants, FRAP assay

## Abstract

*Syzygium cumini* (L.) Skeels (jamun) is an ornamental tree species that is sensitive to salinity. Salinity stress is a major challenge, particularly in regions with saline irrigation water. In the present study, the ameliorative potential of foliar application of an aqueous extract of *Delonix regia* (Poinciana) flowers (PFE) to saline water-irrigated jamun seedlings was investigated over a period of two years. PFE was effective in mitigating the harmful effects of salinity on plant growth, physiology, and biochemistry. Salinity-induced reductions in plant height, leaf area, and biomass which were significantly alleviated by PFE foliar application. The extract also enhanced antioxidant activity, as indicated by increased ferric reducing antioxidant potential (FRAP) and phenolic content, while also reducing hydrogen peroxide (H_2_O_2_) levels and membrane damage as indicated by the accumulation of malondialdehyde (MDA). Additionally, the foliar application of PFE promoted the accumulation of free proline, an essential osmo-protectant, further enhancing the plant’s resilience to salinity stress. These findings highlight the potential of PFE as an eco-friendly bio-stimulant to improve salinity tolerance in jamun and pave the way for sustainable salinity management strategies in other crops as well.

## 1. Introduction

*Syzygium cumini* (L.) Skeels, commonly known as ‘Jamun’ or ‘Java Plum’, is an important plant of the family Myrtaceae [1]. It is valued as an ornamental tree in gardens and for forestry in humid zones, cultivated as a fruit species, and utilized as an important medicinal plant for curing various important human ailments, including diabetes [2]. Therefore, it has been described as an important and promising future fruit [3], to be consumed fresh after harvesting or after processing into value-added products, to increase its shelf life. Moreover, various parts of this plant are used as the key ingredients in the Indian traditional system of medicine. Furthermore, due to several benefits of the plant, it can be effectively used in multipurpose agro-forestry systems [4]. The plant also performs well on marginal lands, where it is considered a suitable tree species due to its capacity to withstand adverse climatic conditions [5]. Since its fruit is an important nutraceutical commodity, its large-scale cultivation in semi-arid environments could contribute to the nutritional security of mankind and a represent an important step toward resource utilization in a sustainable manner.

Jamun is considered sensitive to soil salinity (ECe 7–10 dS m^−1^) under field conditions [6]. However, in an experiment, a variability in salinity stress tolerance was recorded among the 48 genotypes of jamun studied, and it was found that the genotypes with a higher capability of Na^+^ exclusion were tolerant [1]. This experiment also demonstrated that salt stress has an important bearing on the growth and physiology of the plant. Since several methods are being explored for sustainable salt stress management, developing an effective method for salt stress management in this important nutraceutical fruit is essential.

Water scarcity is increasingly limiting economic growth in arid and semi-arid countries. Consequently, compensating for water scarcity requires lower-quality water, such as salt water. Salinity is a major environmental concern for growers due to its detrimental effects on plant growth and agricultural productivity. Osmotic exertion, hormonal imbalances, decreased nutritional incentive, ionic toxicity, and ROS production are some of the various ways in which salts impact plant growth [7,8,9]. Irrigation with saline or low-quality water causes burns and necrosis in the leaves [10,11]. Currently, a variety of strategies for the management of salt stress in plants are under investigation to obtain optimum crop yield [12,13,14,15]. However, the use of bio-stimulants like plant growth-promoting rhizobacteria and plant extracts are considered important in achieving this in an environmentally sustainable manner [16,17,18,19,20].

*Delonix regia* (Boj. ex Hook.) Raf. (syn: *Poinciana regia*), an important flowering tree in the Fabaceae family (commonly known as ‘Poinciana’), is commonly grown in gardens for its bright orange–red flowers. These flowers are rich in bio-active constituents, including antioxidants, carotenoids, polyphenols, and flavonoids [21,22,23], which are important in alleviating environmental stress and reducing ROS accumulation in plants. The use of Poinciana flower extract in the management of biotic stress (root-knot nematode) in tomatoes (PFE) has already been reported [24]; however, not much information is available on its use in the management of abiotic stress, including salinity. Therefore, the present study was conducted to evaluate the effects of PFE foliar application to jamun seedlings as a bio-stimulant for alleviating the adverse effects of salinity, which have never been published. The present study is the first report on the effectiveness of Poinciana flower extract (PFE) in ameliorating salinity stress in jamun seedlings.

## 2. Materials and Methods

### 2.1. Planting Methodology

A pot experiment was conducted in the Ornamental Greenhouse, Horticulture Department, Agriculture Faculty, Tanta University, Tanta, Egypt, from June to November in two consecutive years, 2023 and 2024. The uniform jamun seedlings (6 months old) planted in 20 cm plastic pots filled with clay loam soil were selected for this investigation. The soil analysis showed the following: sand: 67.24%; clay: 21.62%; silt: 11.14%; pH: 7.28; EC: 1.53 dS m^−1^; Ca^+2^: 8.11 meq L^−1^; N: 0.31%; P: 0.045%; and K: 0.07%.

### 2.2. Poinciana Extract (PFE) Preparation and Treatments

Poinciana flowers were collected from trees grown in the Horticulture Department Nursery, Tanta University. For the preparation of Poinciana flower extract (PFE), a custom-built machine was used to blend 450 g of fresh flowers with 900 mL of water (1:2, *w*/*v*). The extract was centrifuged for 10 min and filtered through muslin cloth to obtain stook solution. The PFE solution at 40% concentration, which was determined based on our preliminary experiments, was used for this study. Foliar applications of PFE were performed three times: the first was on 23 June, and two subsequent applications were at 15-day intervals in the morning hours. The untreated plants received a similar foliar spray of tap water thrice, at the same time, as the PFE application for both seasons. The PFE biochemical analysis estimated according to AOAC [25] is presented in Table 1.

### 2.3. Saline Water Treatments

The pots were divided into four groups; the first group was employed as a control and irrigated with tap water, the second group was subjected to salt stress (200 mM NaCl), the third group was foliar sprayed with aqueous extract of PFE, and the fourth was subjected to salt stress along with the foliar spray of PFE. The seedlings were subjected to salt stress on 20 June 2023 and 2024. To prevent them from experiencing osmotic shock, the seedlings were subjected first to saline water at 50 mM, and this concentration was gradually elevated by 50 mM every 3 days, reaching the desired level of 200 mM. The experiment was laid out in a completely randomized design with four treatments and three replicates per treatment. Furthermore, each replicate consisted of five pots.

### 2.4. Growth Parameters

Six pots were picked up from each treatment 63 days from the beginning of the experiment (T1) and 126 days (T2) for growth analysis determination. The plant height, fresh weight of shoot, and root samples was recorded separately, and their dry weights were recorded. The root–weight ratio (g g^−1^) = root mass/total plant biomass, the shoot–weight ratio (g g^−1^) = shoot mass/total plant biomass, and the root:shoot weight ratio was recorded. Leaf area (cm^2^) was estimated by an automatic leaf area meter (Li-3100, LiCor, Lincoln, NE, USA). Crop growth rate (CGR: g m^2^ day^−1^) = (M2 − M1)/q (T2 − T1). The absolute growth rate (AGR: g day^−1^) = (M2 − M1)/(T2 − T1), and the specific leaf area (SLA: cm^2^ g^−1^) = leaf area/leaf weight was also calculated, where M is the biomass and the biomasses M1 and M2 were recorded at the times T1 and T2, respectively.

### 2.5. Physiological and Biochemical Analysis

At 126 days after planting, leaf samples were collected and submerged in liquid nitrogen and then kept at 80 °C for biochemical analysis.

#### 2.5.1. Total Chlorophyll and Carbohydrates

The total chlorophyll was evaluated spectrophotometrically following the method suggested by Dere et al. [26] and expressed in mg g^−1^ FW. The total carbohydrate content in jamun leaves was estimated using the anthrone method [27] and expressed in percentage.

#### 2.5.2. Total Phenols, Proline, and Ferric Reducing Antioxidant Potential (FRAP)

Total phenols (mg GAE kg^−1^ DW) were measured using gallic acid as a standard following the method of Dewanto et al. [28]. Further, the free proline (mg 100 g^−1^ FW) content in jamun leaves was estimated using the Bates [29] procedure. The ferric reducing antioxidant potential (Umol g^−1^ FW) was determined in jamun leaves using the method of Benzie and Strain [30].

#### 2.5.3. Malondialdehyde (MDA) and Hydrogen Peroxide (H_2_O_2_)

Malondialdehyde (MDA) and hydrogen peroxide (H_2_O_2_) were determined in jamun leaves: the MDA content (mg g^−1^ FW) was used to determine the amount of lipid peroxidation according to Heath and Packer [31]. The H_2_O_2_ content (μmol g^−1^ FW) was measured using the method of Patterson et al. [32].

### 2.6. Statistical Design and Data Analysis

The experiment was laid out in a randomized complete design with four treatments, and each treatment was replicated thrice. The collected data were statically analyzed using COSTAT version 6.4 software (CoHort Software, Monterey, CA, USA). Duncan’s test was used to determine significant differences among the means of the treatments at the 5% level of significance. Results are exhibited as the average means ± standard error (SE).

## 3. Results

### 3.1. Growth Parameters

**Figure 1:**
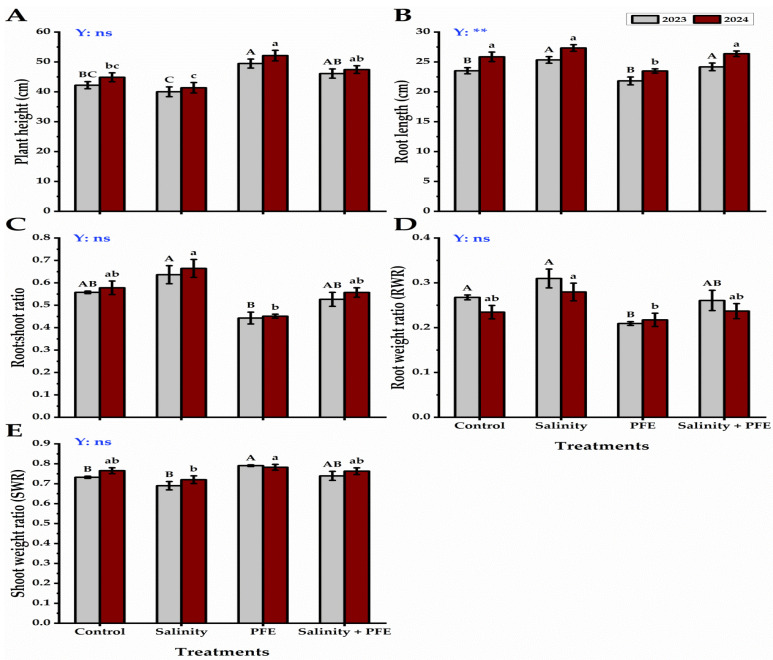
Response of plant height (cm) (**A**), root length (cm) (**B**), root: shoot ratio (**C**), root–weight ratio (g g^−1^) (**D**), and shoot weight ratio (g g^−1^) (**E**) of *Syzygium cumini* L. to Poinciana flower extract (PFE) foliar application under salinity stress for 2023 and 2024 seasons. Bars with the same letters (uppercase for the first season and lowercase for the second season) are not significant at *p* ≤ 0.05 level. Y: means the year, ns: non-significant, ** significant within the two years at *p* ≤ 0.05 and 0.01.

**Figure 2:**
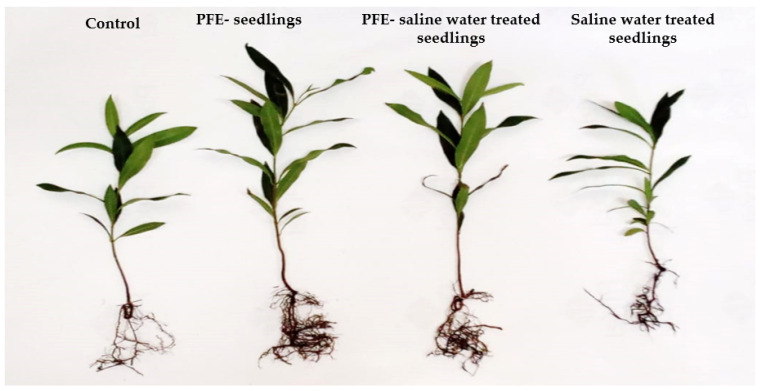
The photograph indicates *Syzygium cumini* L. seedling’s response to Poinciana flower extract (PFE) foliar application under salt stress. Control, PFE seedlings (foliar sprayed seedlings with PFE), PFE-saline water-treated seedlings, and saline water-treated seedlings.

Analysis of variance for the obtained data showed that non-significant differences between the two years were recorded in almost all root traits except root length (Figure 1). The plant height of the jamun seedlings was recorded to be minimum under saline water treatment for two consecutive growing seasons (2023 and 2024); however, the maximum plant height was recorded upon the PFE treatment (Figure 1A). A significant (Duncan’s test, *p* ≤ 0.05) recovery in plant height was noticed in the combined treatment of PFE + saline water rather than for the saline water treatment alone, which was even higher than the height of the control plants (Figure 2).

The root length exhibited the highest values under saline treatments for the two seasons (Figure 1B), but it lowered to the minimum under the PFE treatment. A non-significant reduction in the root length was observed under the saline treatment upon PFE treatment from the saline treatment alone.

The root–shoot ratio was recorded to be the maximum under the saline water treatment; however, it significantly decreased upon the PFE treatment under both growing seasons (Figure 1C). Moreover, a non-significant reduction in the root–shoot ratio was observed in PFE + saline water jamun seedlings from the saline treatment alone, as it was quite similar to that of the control seedlings. The root–weight ratio of jamun seedlings presented the highest values under the saline water treatment for the two growing seasons, but these values reduced to reach the lowest under PFE treatment (Figure 1D). Both PFE + saline water and control seedlings showed a non-significant reduction in the root–weight ratio. On the contrary, the shoot–weight ratio recorded the minimum in jamun seedlings subjected to saline water treatment, and the maximum values were recorded by PFE-treated seedlings for the two seasons (Figure 1E).

**Figure 3:**
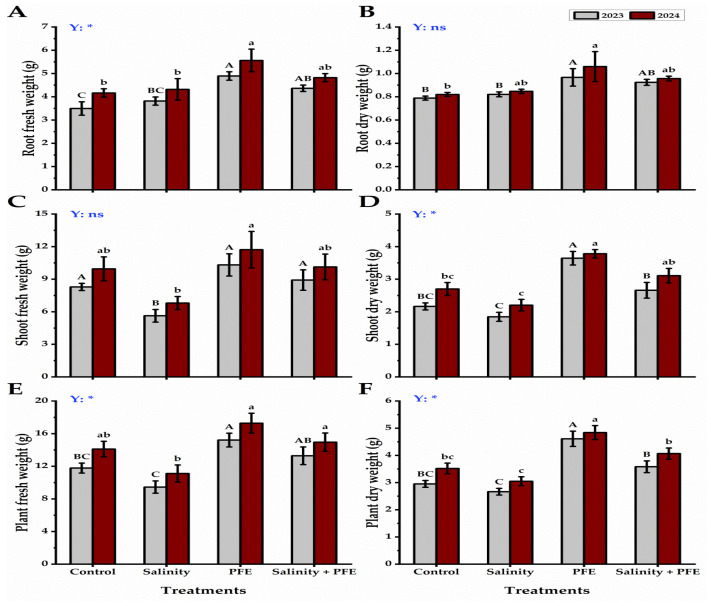
Response of root fresh weight (g) (**A**), root dry weight (g) (**B**), shoot fresh weight (g) (**C**), shoot dry weight (g) (**D**), plant fresh weight (g) (**E**), and plant dry weight (g) (**F**) of *Syzygium cumini* L. to Poinciana flower extract (PFE) foliar application under salinity stress for 2023 and 2024 seasons. Bars with the same letters (uppercase for the first season and lowercase for the second season) are not significant at *p* ≤ 0.05 level. Y: means the year, ns: non-significant, * significant within the two years at *p* ≤ 0.05.

The lowest root fresh and dry weights were exhibited by control seedlings, and there was a non-significant change with saline seedlings during the two consecutive years, while the PFE treatment alone had the highest root fresh weight (Figure 3A,B). On the other hand, the highest shoot fresh and dry weights were observed in the seedlings foliar sprayed with PFE, followed by PFE + saline water seedlings, while the lowest values were recorded by the saline water-treated seedlings (Figure 3C,D). The plant’s fresh and dry weight was lowered to the minimum in the saline water-treated seedlings of jamun, followed by the control seedlings, which were increased further in the seedlings subjected to PFE + saline water. The maximum plant fresh and dry weight was observed in seedlings treated by PFE alone during two consecutive years of observation (Figure 3E,F).

**Figure 4:**
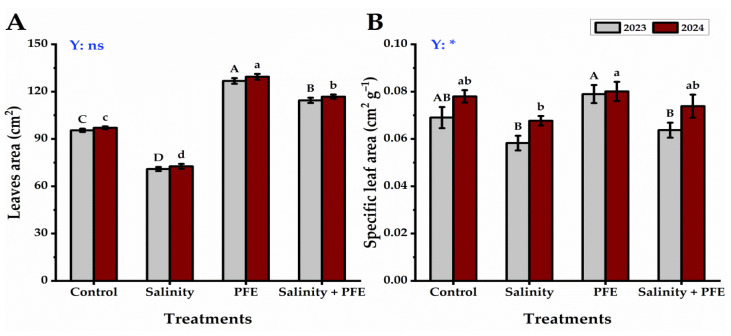
Response of leaves area (cm^2^) (**A**) and specific leaf area (cm^2^ g^−1^) (**B**) of *Syzygium cumini* L. to Poinciana flower extract (PFE) foliar application under salinity stress for 2023 and 2024 seasons. Bars with the same letters (uppercase for the first season and lowercase for the second season) are not significant at *p* ≤ 0.05 level. Y: means the year, ns: non-significant, * significant within the two years at *p* ≤ 0.05.

Saline water treatments showed a great reduction in the leaf traits of jamun seedlings, as leaf area showed the lowest in saline water-treated seedlings, while the highest leaf area values were given by PFE seedlings (Figure 4A). The SLA of jamun seedlings showed the lowest values under both treatments of saline water and PFE + saline water, followed by the control, during the two consecutive years (Figure 4B). On the contrary, PFE-treated seedlings presented the highest values in this respect.

**Figure 5:**
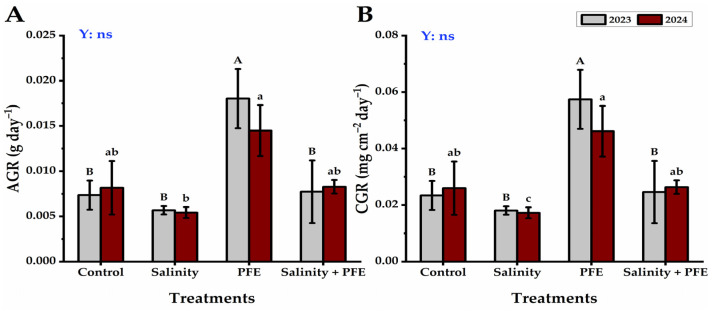
Response of absolute growth rate (g day^−1^) (**A**) and crop growth rate (mg cm^−2^ day^−1^) (**B**) of *Syzygium cumini* L. to Poinciana flower extract (PFE) foliar application under salinity stress for 2023 and 2024 seasons. Bars with the same letters (uppercase for the first season and lowercase for the second season) are not significant at *p* ≤ 0.05 level. Y: means the year, ns: non-significant within the two years at *p* ≤ 0.05.

The AGR of jamun seedlings was lowest under the saline water treatment, followed by the control seedlings, which improved further under the PFE + saline water treatment; however, the highest AGR was observed under the PFE treatment alone during two consecutive years (Figure 5A). The CGR recorded the lowest rate in the saline water-treated seedlings, followed by the control seedlings, which improved further under the PFE + saline water treatment; however, the CGR was increased under the PFE treatment alone during the two consecutive years (Figure 5B).

### 3.2. Physiological and Biochemical Analysis

#### 3.2.1. Total Chlorophyll and Carbohydrates Content

**Figure 6:**
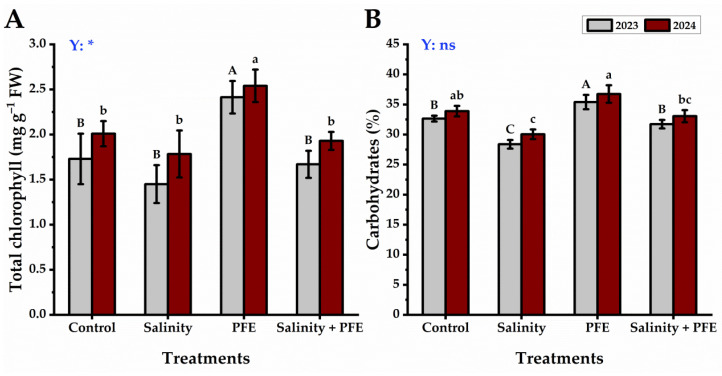
Response of total chlorophyll (**A**) and carbohydrates (**B**) content of *Syzygium cumini* L. to Poinciana flower extract (PFE) foliar application under salinity stress for 2023 and 2024 seasons. Bars with the same letters (uppercase for the first season and lowercase for the second season) are not significant at *p* ≤ 0.05 level. Y: means the year, ns: non-significant, * significant within the two years at *p* ≤ 0.05.

The total chlorophyll content of the jamun leaves was recorded to be the lowest in saline water-treated seedlings, which was statistically not significantly different from either PFE + salinity or control seedlings; however, the highest total chlorophyll content was observed in the PFE treatment alone, which was significantly higher than all other treatments in the two connective seasons (Figure 6A).

On the other hand, the total carbohydrate content was found to be lowest in the saline water-treated seedlings, followed by the PFE + salinity treatment and control, while the highest total carbohydrate content was observed in the PFE treatment alone in the two connective seasons (Figure 6B).

#### 3.2.2. Total Phenols, Proline, and FRAP Content

**Figure 7:**
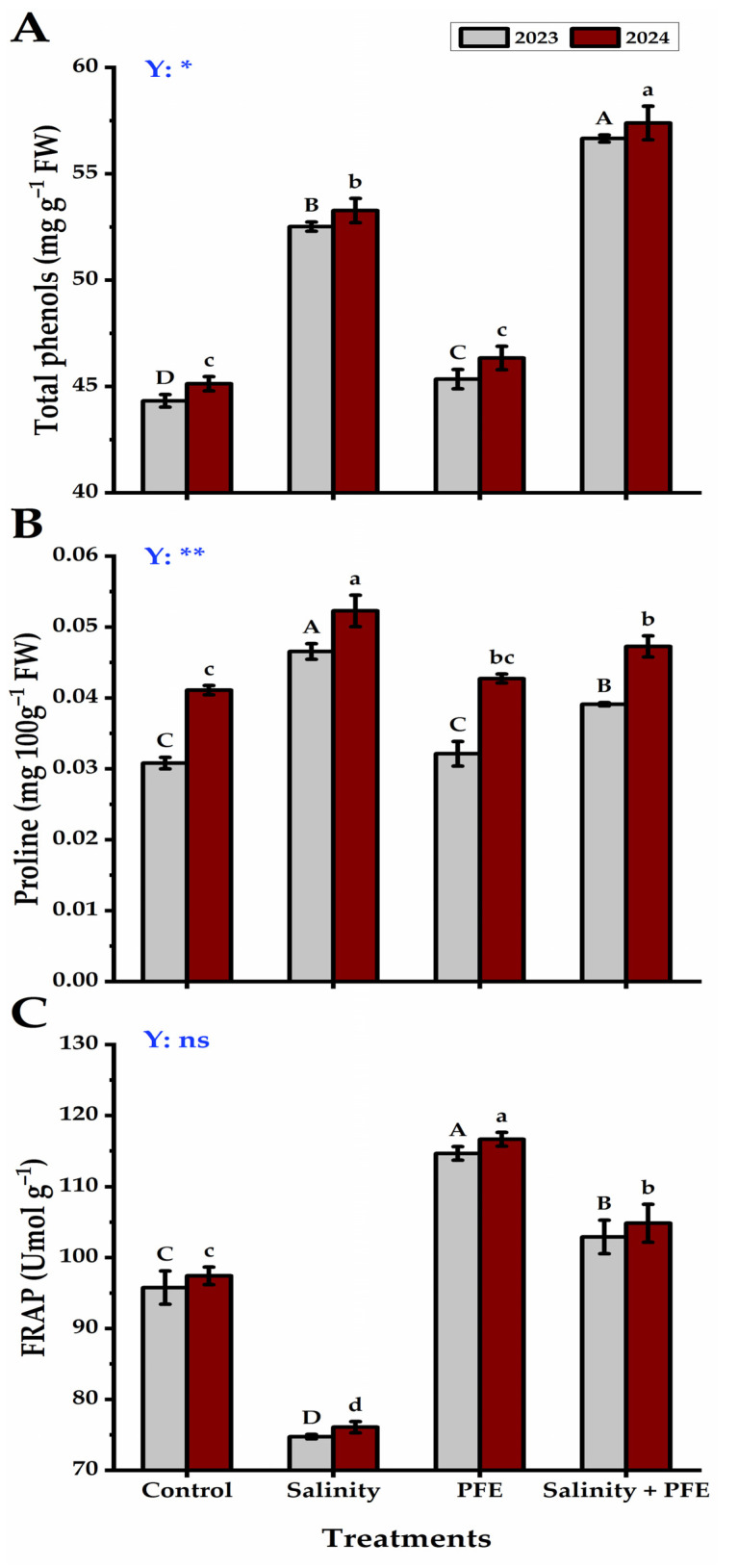
Response of total phenols (**A**), proline (**B**), and FRAP (**C**) content of *Syzygium cumini* L. to Poinciana flower extract (PFE) foliar application under salinity stress for 2023 and 2024 seasons. Bars with the same letters (uppercase for the first season and lowercase for the second season) are not significant at *p* ≤ 0.05 level. Y: means the year, ns: non-significant, * significant within the two years at *p* ≤ 0.05. ** significant within the two years at *p* ≤ 0.05 and 0.01.

The total phenols of the jamun seedlings were recorded to be significantly lower in control than in all other treatments (Figure 7A). An increase in total phenols was noticed in saline water-treated seedlings, while the maximum phenol value was given by PFE + salinity seedlings. The accumulation of free proline was more in the saline water-treated seedlings than in the control and PFE-treated seedlings (Figure 7B); however, it decreased significantly when saline water treated seedlings received PFE foliar application. The lowest proline values were noticed in control and PFE-treated seedlings with non-significant differences among them in both seasons. On the contrary to proline accumulation, there was a significant reduction in the FRAP of the saline water-treated seedlings relative to the control and other treated seedlings; however, the amelioration of salinity stress using PFE further increased significantly to reach the maximum level under the PFE treatment alone in the two consecutive seasons (Figure 7C).

#### 3.2.3. MDA and H_2_O_2_

**Figure 8:**
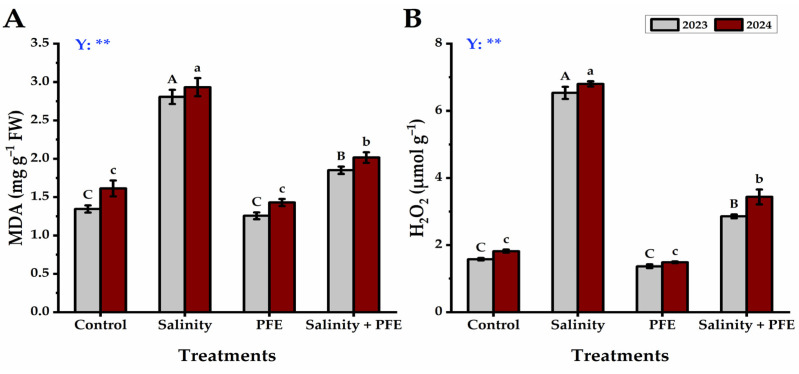
Response of MDA (**A**) and H_2_O_2_ (**B**) content of *Syzygium cumini* L. to Poinciana flower extract (PFE) foliar application under salinity stress for 2023 and 2024 seasons. Bars with the same letters (uppercase for the first season and lowercase for the second) are not significant at *p* ≤ 0.05 level. Y: means the year, ns: non-significant, ** significant within the two years at *p* ≤ 0.05 and 0.01.

The MDA content of the jamun seedlings reached the highest under salinity stress, which was reduced significantly in PFE+ salinity seedlings; however, it was further reduced under PFE treatment alone, compared to the control, in two consecutive years of experimentation (Figure 8A). In a similar manner, the H_2_O_2_ content of the saline water-treated seedlings was found to be maximum (Figure 8A), which was significantly reduced under PFE-mediated salinity stress amelioration (2.86 ± 0.06 and 3.44 ± 0.22 µmol g^−1^, respectively); however, it was further reduced under PFE treatment alone, and it was comparable to the control with non-significant differences among them in two consecutive years of experimentation.

### 3.3. Correlation Analysis

**Figure 9:**
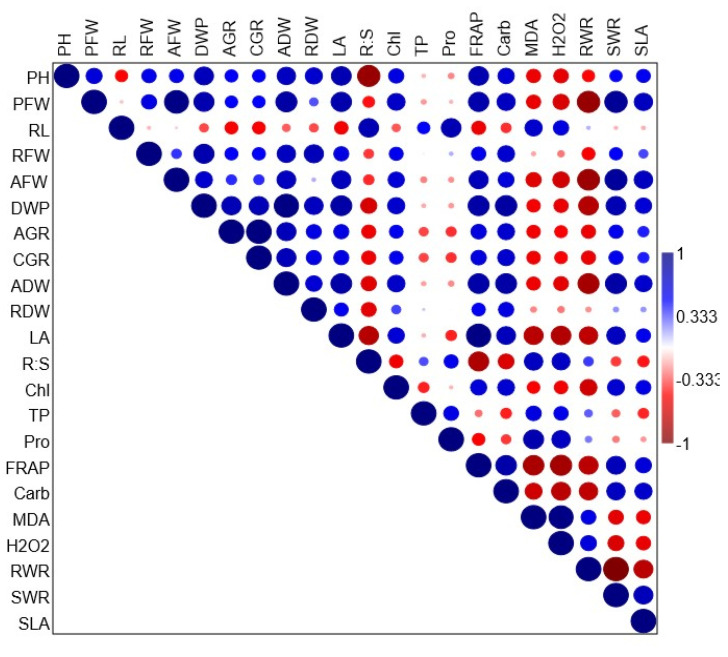
The correlation plot represents Pearson’s correlation analysis between growth traits. The size of the circles is proportional to the absolute value of correlation coefficients, whereas their color represents the value in positive or negative correlation region as represented in the right bar of varying color intensities from intense blue to intense red, ranging from 1 to −1, respectively. Plant height (PH), plant fresh weight (PFW), root fresh weight (RFW), shoot fresh weight (AFW), plant dry weight (DWP), shoot dry weight (ADW), root dry weight (RDW), leaf area (LA), FRAP, carbohydrates (Carb), shoot–weight ratio (SWR), specific leaf area (SLA), AGR, CGR, total chlorophyll (Chl), root:shoot ratio (R:S), total phenol content (TP), protein (Pro), MDA, H_2_O_2_, root weight ratio (RWR).

The correlation plot in Figure 9 shows the results of Pearson’s correlation analysis between estimated growth and biochemical traits affected by PEF foliar application for saline water-treated seedlings. Plant height, plant fresh weight, root fresh weight, shoot fresh weight, plant dry weight, shoot dry weight, root dry weight, leaf area, FRAP, carbohydrates, shoot–weight ratio, and SLA levels were all strongly positively correlated. On the other hand, AGR, CGR, and total chlorophyll show weak positive correlations. Moreover, the root:shoot ratio, total phenols, protein, MDA, H_2_O_2_, root weight ratio, and levels exhibited clear negative correlations.

## 4. Discussion

The growth, development, and productivity of plants are restricted by many environmental conditions including saline water. Saline water irrigation is a major factor that causes soil salinity. The rises in soil salt concentration caused osmotic effects, soil water potential falling, inhibiting root water absorption and ultimately water deficit [33,34,35]. Additionally, salt stress decreases cell turgor and development [36,37]. Furthermore, stomatal conductance is negatively impacted by water scarcity, which reduces biomass production and carbon absorption and fixation [38,39]. Many reports have stated that salt stress can be ameliorated using bio-stimulants [40,41].

### 4.1. Vegetative Growth

Various bio-stimulants including plant extract-based bio-stimulants are known to effectively manage salinity stress in plants [42,43]. Since the flowers of Poinciana (*Delonix regia*) are produced in abundance and are a rich source of natural antioxidants which may have a variety of applications [44], it was anticipated that they may possess similar properties of plant stress amelioration and can be effectively utilized for the management of salinity stress in plants. Poinciana flowers are a rich source of several bio-active compounds [21,22,23] and therefore could be a suitable candidate for plant protection inventions. Its usefulness in the management of root-knot nematode in tomatoes has already been suggested [24]. However, no detailed information is available on the effect of that flower extract on the amelioration of abiotic stresses. In the present study, saline water-treated seedlings showed a significant reduction in plant growth, while the PFE foliar application significantly produced an improvement in the growth, development, and metabolites and lowered the harmful impacts of the oxidative stress in jamun seedlings grown under saline conditions.

Poinciana flowers have a lot of active substances, including flavanoids, terpenoid saponins, phenolic compounds, carbohydrates, tannins and glycosides [45], which serve to enhance the plant’s growth. Treated seedlings with PFE foliar application exhibited the maximum plant height, leaf area, and leaf weight ratio. Leaf area is an important indicator of plant growth and photosynthetic capacity. In the present study, when the leaf area was measured, it was found that there was a clear-cut statistically significant reduction in leaf area under salinity, which was significantly improved upon PFE treatment. Leaf area presents a slight plant response to PFE treatment. The PFE’s role in promoting vegetative growth may be revealed by the hypothesis that PFE contains important active substances e.g., terpenoid, the precursor of gibberellic acid [46], which is crucial for the plant growth [47]. The high content of N in PFE caused maximum plant growth, as N induces nutrient uptake in plants [48,49,50]. The foliar application of PFE lowered the negative effect of salinity in jamun seedlings, inducing an increase in the root:shoot ratio and root–weight ratio.

An enhancement was observed in the growth analysis values of AGR and CGR of PFE and PFE + saline water-treated seedlings. Maximum CGR refers to the highest possible conversion rates and biomass output potential. Poinciana flowers contain many active ingredients, including amino acids, proteins, and carbohydrates [22], which are important in inducing plant growth. The improvements detected in the growth and biomass confirm our hypothesis that PFE can ameliorate the negative effects of saline water on the growth and development of jamun seedlings.

### 4.2. Root Growth

An increase in the root traits of length, fresh and dry weights in saline water-treated seedlings was observed as compared with control seedlings. Under salt stress, plant roots alter morphologically in length, thickness, quantity, and proliferation; also, the root density and electrical conductivity rise; all these factors are useful to improve the water absorption and nutrition under salinity [51]. Additionally, the growth of roots helps plants accumulate fewer toxic ions [41]. Foliar spraying with PFE produced the lowest root length, but these were thicker than the other treatments, as shown in the root weights (Figure 1). According to Drew and Saker [52], physiological reactions often take place prior to morphological ones. It indicates that a rise in ion absorption through foliar spray might serve as a sign to start new roots and increase root biomass.

### 4.3. Biochemical Analysis

A significant increase in chlorophyll content was noticed following PFE foliar application as compared with saline water-treated seedlings, but no major changes were noticed upon other treatments. PFE is rich in Mg, which has a vital role in chlorophyll formation [53,54]. In this study, the observed pattern of photosynthetic pigments remained consistent with the carbohydrate content, showing the major effects of PFE treatments. Under saline conditions, the carbohydrates generated in plant cells have an osmo-protective function in enhancing photosynthesis and water and nutrient absorption, which are all necessary to boost metabolic processes [55]. The elevated carbohydrates content in PFE (Table 1) may be attributed to the total carbohydrates in jamun plant seedlings with PFE and PFE + salinity treatments. Furthermore, increased carbohydrate content may result from improved photosynthetic pigments after PFE treatment. According to this study, the increase in the overall carbohydrate content of jamun leaves after PFE treatments indicated increased yield and growth and increased metabolite synthesis and accumulation.

A sharp increase in the FRAP of the PFE-saline water-treated seedlings than the saline water-treated seedlings was observed. Furthermore, the phenolic content (a class of cellular antioxidants) also increased significantly in the PFE-saline water-treated seedlings compared with the saline water-treated seedlings. The FRAP assay is a method that measures the antioxidant activity of any substance by measuring its ability to reduce Fe^3+^ to Fe^2+^. The increased antioxidant status may be attributed to the fact that the flowers of poinciana are a rich source of natural antioxidants including phenolics, additionally, poinciana flowers extract presented IC50 lower than 50 μg mL^−1^, which is a very strong antioxidant [22,44], and therefore, they could be helpful in promoting the antioxidant status of PFE-treated seedlings under salt stress. This increase may collaborate with the commonly occurring oxidative burst during the salt stress [56,57] as indicated by the enhanced H_2_O_2_ level under the saline condition in the present study. Further, we also noticed a clear and significant decline in H_2_O_2_ content in PFE-treated seedlings under salinity in comparison to the plants under salinity stress. Such an increase in ROS under salinity leads to the damage of the membrane, leading to an enhanced accumulation of MDA in the plant tissue [58,59]. In the present study, the salinity-induced accumulation of MDA was significantly reduced by the foliar application of PFE in the Jamun seedlings under salinity stress.

Proline is an important osmo-protectant, and its accumulation is linked to improving the ability to cope with various environmental stresses, including salinity [60,61]. In the present study, the salinity-induced increase in free proline was further accumulated under the foliar application of PFE. Therefore, the enhanced accumulation of free proline could be linked to improving the capability of jamun seedlings to withstand the salinity stress. Therefore, the present study provides conclusive evidence that the foliar application of the aqueous extract of poinciana flowers on jamun seedlings acts as an organic bio-stimulant for promoting plant growth by alleviating the adverse effects of salinity. Poinciana flower extract not only promotes the antioxidant status of salinity-affected jamun seedlings but also reduces the accumulation of ROS and membrane damage, as well as accumulates free proline, which is an important osmo-protectant.

## 5. Conclusions and Future Prospects

As a result, poinciana flower extract (PFE) as an environmentally safe plant growth stimulant may enhance the growth parameters and photosynthetic pigments of jamun seedlings that were reflected in enhancing the productivity, secondary metabolites, and antioxidant activity as well as increased plant resistance of jamun seedlings grown under saline conditions. PFE is an outstanding source of nutrients, phytohormones, phenolics, and antioxidants. To control the development and production of tree plants under saline conditions, PFE can be employed as an alternative and environmentally safe plant growth stimulant. The use of poinciana flower extract can be established as a common bio-stimulant with its widespread utilization only after detailed studies on a variety of stresses on a wide range of crop plants on a case-by-case basis in various crop–stress type combinations.

## Figures and Tables

**Table 1:** Proximate analysis of *Poinciana regia* Hook. flower extract.

Proximate Analysis	Moisture %	Ash %	Protein %	Fiber **%**	Fat **%**	Carbohydrates %	Total Phenols mg g^−1^	Total Flavonoids mg g^−1^	DPPH (IC50) **μg mL**^−1^	P mg 100 g^−1^	**K** mg 100 g^−1^	Ca mg 100 g^−1^	Mg mg 100 g^−1^
Values	10.35	2.11	11.32	13.82	12.76	49.64	32.50	30.1	33.72	4.11	8.25	2.34	5.66

## Data Availability

The original contributions presented in the study are included in the article, further inquiries can be directed to the corresponding author.

## References

[1] Singh A., Kumar A., Kumar R., Prakash J., Kumar N., Verma A.K. (2024). Evaluation of salt tolerance in jamun (*Syzygium cumini* L. Skeels) using morpho-physiological traits and membership function analysis. Sci. Hortic..

[2] Ayyanar M., Subash-Babu P. (2012). *Syzygium cumini* (L.) Skeels: A review of its phytochemical constituents and traditional uses. Asian Pac. J. Trop. Biomed..

[3] Madani B., Mirshekari A., Yahia E.M., Golding J.B., Hajivand S., Dastjerdy A.M. (2021). Jamun (*Syzygium cumini* L. Skeels): A promising fruit for the future. Hortic. Rev..

[4] Sarvade S., Gautam D.S., Bhalawe S., Bisen P.K. (2016). An overview of potential multipurpose agroforestry tree species, *Syzygium cuminii* (L.) Skeels in India. J. Appl. Nat. Sci..

[5] Tewari S.K., Singh D., Nainwal R.C., Nair K.N. (2017). Horticultural management of *Syzygium cumini*: *Syzygium cumini* and other underutilized species. The Genus Syzygium.

[6] Singh G., Lal K. (2018). Review and case studies on biodrainage: An alternative drainage system to manage waterlogging and salinity. Irrig. Drain..

[7] Kumar A., Singh S., Gaurav A.K., Srivastava S., Verma J.P. (2020). Plant growth-promoting bacteria: Biological tools for the mitigation of salinity stress in plants. Front. Microbiol..

[8] Berni R., Luyckx M., Xu X., Legay S., Sergeant K., Hausman J.F., Lutts S., Cai G., Guerriero G. (2019). Reactive oxygen species and heavy metal stress in plants: Impact on the cell wall and secondary metabolism. Environ. Exp. Bot..

[9] Ahmad P., Nabi G., Jeleel C.A., Umar S., Ahmad P., Umar S. (2011). Free radical production, oxidative damage and antioxidant defense mechanisms in plants under abiotic stress. Oxidative Stress: Role of Antioxidants in Plants.

[10] Rasool S., Hameed A., Azooz M.M., Siddiqi T.O., Ahmad P. (2013). Salt stress: Causes, types and responses of plants. Ecophysiology and Responses of Plants Under Salt Stress.

[11] Al-Hattab Z.N., Al-Ajeel S.A., El-Kaaby E.A. (2015). Effect of salinity stress on *Capsicum annuum* callus growth, regeneration and callus content of capsaicin, phenylalanine, proline and ascorbic acid. J. Life Sci..

[12] Krishnamurthy L., Serraj R., Hash C.T., Dakheel A.J., Reddy B.V.S. (2007). Screening sorghum genotypes for salinity tolerant biomass production. Euphytica.

[13] Veeru P., Kishor M.P., Meenakshi M. (2009). Screening of medicinal plant extracts for antioxidant activity. J. Med. Plant Res..

[14] Sadat-Hosseini M., Naeimi A., Boroomand N., Aalifar M., Farajpour M. (2022). Alleviating the adverse effects of salinity on Roselle plants by green synthesized nanoparticles. Sci. Rep..

[15] Romero-Aranda R., Soria T., Cuartero J. (2001). Tomato plant-water uptake and plant-water relationships under saline growth conditions. Plant Sci..

[16] Sharma I., Sharma A., Bhardwaj R., Sirhindi G. (2023). PGPR (Plant Growth Promoting Rhizobacteria) for Plant Stress Management.

[17] Singh N., Maurya V., Singh H., Sharma S., Sharma I., Kumar R., Sharma A. (2024). Salinity stress in crop plants: Effects and eco-friendly management. Adv. Food Secur. Sustain..

[18] Porcel R., Aroca R., Ruiz-Lozano J.M. (2012). Salinity stress alleviation using arbuscular mycorrhizal fungi. A review. Agron. Sustain. Dev..

[19] Paul D., Lade H. (2014). Plant-growth-promoting rhizobacteria to improve crop growth in saline soils: A review. Agron. Sustain. Dev..

[20] Azooz M.M., Metwally A., Abou-Elhamd M.F. (2015). Jasmonate-induced tolerance of Hassawi okra seedlings to salinity in brackish water. Acta Physiol. Plant..

[21] Adjé F.A., Lozano Y.F., Le Gernevé C., Lozano P.R., Meudec E., Adima A.A., Gaydou E.M. (2012). Phenolic acid and flavonol water extracts of *Delonix regia* red flowers. Ind. Crop. Prod..

[22] Shabir G., Anwar F., Sultana B., Khalid Z.M., Afzal M., Khan Q.M., Ashrafuzzaman M. (2011). Antioxidant and antimicrobial attributes and phenolics of different solvent extracts from leaves, flowers and bark of Gold Mohar [*Delonix regia* (Bojer ex Hook.) Raf.]. Molecules.

[23] Das A.K., Kashyap K., Bhardwaj A.K., Biswas S., Roymahapatra G., Bhattacharyya S., Hait M. (2024). Proximate Analysis, and Mineral Content Determination of *Delonix regia* Flower. ES Food Agrofor..

[24] Adamu M.Y., Peter P., Yusuf M.I. (2023). Effect of flamboyant flower (*Delonix regia*) powder on root knot nematodes (*Meloidogyne incognita*) infestation on tomato plant (*Solanum lycopersicum*) in Yola, Adamawa State. Bio-Research.

[25] AOAC Official Methods of Analysis. Proceedings of the 17th Edition, Association of Official Analytical Chemists.

[26] Dere S., Güne¸s T., Sivaci R. (1998). Spectrophotometric determination of chlorophyll-A, B and total carotenoid contents of some algae species using different solvents. Turk. J. Bot..

[27] Yemm E.W., Willis A.J. (1954). The estimation of carbohydrates in plant extracts by anthrone. Biochem. J..

[28] Dewanto V., Wu X., Adom K.K., Liu R.H. (2002). Thermal processing enhances the nutritional value of tomatoes by increasing total antioxidant activity. J. Agric. Food Chem..

[29] Bates L.S., Waldren R.P., Teare I.D. (1973). Rapid determination of free proline for water-stress studies. Plant Soil.

[30] Benzie I.F., Strain J.J. (1996). The ferric reducing ability of plasma (FRAP) as a measure of “antioxidant power”: The FRAP assay. Anal. Biochem..

[31] Heath R.L., Packer L. (1968). Photoperoxidation in isolated chloroplasts. I. Kinetics and stoichiometry of fatty acid peroxidation. Arch. Biochem. Biophys..

[32] Patterson B.D., Macrae E.A., Ferguson I.B. (1984). Estimation of hydrogen peroxide in plant extracts using titanium (IV). Anal. Biochem..

[33] Pardo J.M. (2010). Biotechnology of water and salinity stress tolerance. Curr. Opin. Biotechnol..

[34] Roy S.J., Negrão S., Tester M. (2014). Salt resistant crop plants. Curr. Opin. Biotechnol..

[35] Meng Y., Yin Q., Yan Z., Wang Y., Niu J., Zhang J., Fan K. (2020). Exogenous silicon enhanced salt resistance by maintaining K^+^/Na^+^ homeostasis and antioxidant performance in alfalfa leaves. Front. Plant Sci..

[36] Munns R. (2005). Genes and salt tolerance: Bringing them together. New Phytol..

[37] Munns R., Tester M. (2008). Mechanisms of salinity tolerance. Annu. Rev. Plant Biol..

[38] Almeida D.M., Oliveira M.M., Saibo N.J.M. (2017). Regulation of Na^+^ and K^+^ homeostasis in plants: Towards improved salt stress tolerance in crop plants. Genet. Mol. Biol..

[39] Ghanem K.Z., Hasham M.M., El-Sheshtawy A.N.A., El-Serafy R.S., Sheta M.H. (2022). Biochar stimulated actual evapotranspiration and wheat productivity under water deficit conditions in sandy soil based on non-weighing lysimeter. Plants.

[40] El-Serafy R.S., El-Sheshtawy A.N.A., Atteya A.K., Al-Hashimi A., Abbasi A.M., Al-Ashkar I. (2021). Seed priming with silicon as a potential to increase salt stress tolerance in *Lathyrus odoratus*. Plants.

[41] El-Serafy R.S., Dahab A.A., Ghanem K.Z., Elhakem A., Bahgat A.R., Venkatesh J., El-Sheshtawy A.A., Badawy A. (2024). As a natural antioxidant: Sesbania Grandiflora leaf extract enhanced growth and yield performance, active ingredients and tolerance of *Hibiscus sabdariffa* L. under salt-affected soil. J. Soil Sci. Plant Nutr..

[42] Ahmad A., Blasco B., Martos V. (2022). Combating salinity through natural plant extracts based biostimulants: A review. Front. Plant Sci..

[43] Arif Y., Sami F., Siddiqui H., Bajguz A., Hayat S. (2020). Salicylic acid in relation to other phytohormones in plant: A study towards physiology and signal transduction under challenging environment. Environ. Exp. Bot..

[44] Jyothi M.V., Narayan M.S., Kotamballi N., Bhagyalakshmi N. (2007). Antioxidative efficacies of floral petal extracts of *Delonix regia* Rafin. Int. J. Biomed. Pharm. Sci..

[45] Hait M., Nemu S.C., Kashyap N.K., Chaturwedi A. (2018). Physicochemical and phytochemical exploration on flower of *Delonex regia*. J. Med. Plants Stud..

[46] Aulya N.R., Supriyatin, Hartanti E.P., Nada W.A.Q., Achmad Syahputa A. (2020). Effect of aqueous and ethanol extract of *Acacia nilotica* L. leaves on seed germination of *Vigna radiata* L.. Indones. J. Sci. Educ..

[47] Gupta R., Chakrabarty S.K. (2013). Gibberellic acid in plant: Still a mystery unresolved. Plant Signal Behav..

[48] Nurzyńska-Wierdak R. (2013). Does mineral fertilization modify essential oil content and chemical composition in medicinal plants?. Acta Sci. Pol. Hortorum. Cultus.

[49] Sakr W.R., El-Sayed A.A., Hammouda A.M., Saad El-Deen F.S.A. (2018). Effect of NPK, aloe gel and moringa extracts on geranium plants. J. Hortic. Sci. Ornam. Plants.

[50] Atteya A.K.G., El-Serafy R.S., El-Zabalawy K.M., Elhakem A., Genaidy E.A.E. (2022). Brassinolide maximized the fruit and oil yield, induced the secondary metabolites, and stimulated linoleic acid synthesis of *Opuntia ficus*-indica oil. Horticulturae.

[51] Acosta-Motos J.R., Ortuño M.F., Bernal-Vicente A., Diaz-Vivancos P., Sanchez Blanco M.J., Hernandez J.A. (2017). Plant responses to salt stress: Adaptive mechanisms. Agronomy.

[52] Drew M.C., Saker L.R. (1978). Nutrient supply and the growth of the seminal root system in barley. III. Compensatory increases in growth of lateral roots, and in rates of phosphate uptake in response to a localized supply of phosphate. J. Exp. Bot..

[53] Taiz L., Zeiger E. (2010). Plant Physiology.

[54] Sheha A.M., Abou El-Enin M.M., El-Hashash E.F., Rady M.M., El-Serafy R.S., Shaaban A. (2023). The productivity and overall benefits of faba bean-sugar beet intercropping systems interacted with foliar applied nutrients. J. Plant Nutr..

[55] Zali A.G., Ehsanzadeh P. (2018). Exogenously applied proline as a tool to enhance water use efficiency: Case of fennel. Agric. Water Manag..

[56] Gémes K., Kim Y.J., Park K.Y., Moschou P.N., Andronis E., Valassaki C., Roussis A., Roubelakis-Angelakis K.A. (2016). An NADPH-oxidase/polyamine oxidase feedback loop controls oxidative burst under salinity. Plant Physiol..

[57] Kaur G., Sharma A., Guruprasad K., Pati P.K. (2014). Versatile roles of plant NADPH oxidases and emerging concepts. Biotechnol. Adv..

[58] Hnilickova H., Kraus K., Vachova P., Hnilicka F. (2021). Salinity stress affects photosynthesis, malondialdehyde formation, and proline content in *Portulaca oleracea* L.. Plants.

[59] Eraslan F., Inal A., Savasturk O., Gunes A. (2007). Changes in antioxidative system and membrane damage of lettuce in response to salinity and boron toxicity. Sci. Hortic..

[60] Liang X., Zhang L., Natarajan S.K., Becker D.F. (2013). Proline mechanisms of stress survival. Antioxid. Redox Signal..

[61] Shafi A., Zahoor I., Mushtaq U., Akhtar M.S. (2019). Proline accumulation and oxidative stress: Diverse roles and mechanism of tolerance and adaptation under salinity stress. Salt Stress, Microbes, and Plant Interactions: Mechanisms and Molecular Approaches.

